# Supplementary material for: Beta-Glucan from S. cerevisiae Protected AOM-Induced Colon Cancer in cGAS-Deficient Mice Partly through Dectin-1-Manipulated Macrophage Cell Energy
Source: Int J Mol Sci. 2022 Sep 19;23(18):10951. doi: 10.3390/ijms231810951 (PMC9505986; doi:10.3390/ijms231810951)
Supplement: Supplementary file 1 [file ijms-23-10951-s001.zip › ijms-1897051-supplementary.pdf]

**Table S1.** The exact p values of the figure with significant statistical analysis are demonstrated.

| | WT vs. cGAS-/- | WT+S. cerevisiae vs. cGAS-/- | cGAS-/-+S. cerevisiae vs. cGAS-/- |
|---|---|---|---|
| Fig 1C | 0.0132 | 0.0132 | 0.0132 |
| Fig 1D | 0.0129 | 0.0129 | 0.0129 |
| | | | |
| Fig 2G Rikennellaceae | 0.003 | 0.0016 | 0.0043 |
| Fig 2G Turicibacteraceae | 0.0014 | 0.0089 | 0.0015 |
| | Control vs yeast extract | Control vs WGP | |
| Fig 3B | 0.0008 | 0.0002 | |
| Fig 3C | 0.0315 | 0.0271 | |
| Fig 3D | 0.0314 | 0.011 | |
| | | | |
